# Interleukin-6 Is a Circulating Prognostic Biomarker for Hepatocellular Carcinoma Patients Treated with Combined Immunotherapy

**DOI:** 10.3390/cancers14040883

**Published:** 2022-02-10

**Authors:** Yuta Myojin, Takahiro Kodama, Ryotaro Sakamori, Kazuki Maesaka, Takayuki Matsumae, Yoshiyuki Sawai, Yasuharu Imai, Kazuyoshi Ohkawa, Masanori Miyazaki, Satoshi Tanaka, Eiji Mita, Seiichi Tawara, Takayuki Yakushijin, Yasutoshi Nozaki, Hideki Hagiwara, Yuki Tahata, Ryoko Yamada, Hayato Hikita, Tomohide Tatsumi, Tetsuo Takehara

**Affiliations:** 1 Department of Gastroenterology and Hepatology, Osaka University Graduate School of Medicine, Suita 565-0871, Japan; myojin@gh.med.osaka-u.ac.jp (Y.M.); t-kodama@gh.med.osaka-u.ac.jp (T.K.); sakamori@gh.med.osaka-u.ac.jp (R.S.); k.maesaka@gh.med.osaka-u.ac.jp (K.M.); a0023092@gh.med.osaka-u.ac.jp (T.M.); yuki.tahata@gh.med.osaka-u.ac.jp (Y.T.); ryo726@gh.med.osaka-u.ac.jp (R.Y.); hikita@gh.med.osaka-u.ac.jp (H.H.); tatsumit@gh.med.osaka-u.ac.jp (T.T.); 2 Department of Gastroenterology and Hepatology, Ikeda Municipal Hospital, Ikeda 563-0025, Japan; yoshiyuki-sawai@hosp.ikeda.osaka.jp (Y.S.); yasuimai@hosp.ikeda.osaka.jp (Y.I.); 3 Department of Hepatobiliary and Pancreatic Oncology, Osaka International Cancer Institute, Osaka 541-8567, Japan; kazuyoshi.ohkawa@oici.jp; 4 Department of Gastroenterology and Hepatology, Osaka Police Hospital, Osaka 543-0035, Japan; mmiya1216@oph.gr.jp; 5 Department of Gastroenterology and Hepatology, National Hospital Organization Osaka National Hospital, Osaka 540-0006, Japan; tanaka.satoshi.eg@mail.hosp.go.jp (S.T.); mita.eiji.zf@mail.hosp.go.jp (E.M.); 6 Department of Gastroenterology and Hepatology, Osaka General Medical Center, Osaka 558-8558, Japan; twr@gh.opho.jp (S.T.); yakushijin@gh.opho.jp (T.Y.); 7 Department of Gastroenterology and Hepatology, Kansai Rosai Hospital, Amagasaki 660-8511, Japan; noza0211@yahoo.co.jp (Y.N.); hagiwara-hideki@kansaih.johas.go.jp (H.H.)

**Keywords:** multiplex immunoassay, immune checkpoint inhibitor, anti-VEGF antibody, anti-PD-L1 antibody, liver cancer

## Abstract

**Simple Summary:**

Hepatocellular carcinoma (HCC) is a major cause of cancer death worldwide. Due to its high recurrence rate, many HCC patients progress to an advanced stage and require systemic therapy. Among six available chemotherapy regimens for advanced HCC, atezolizumab/bevacizumab (Atezo/Bev) combination therapy is considered as a front-line therapy, but approximately 20% of patients are non-responders. Therefore, biomarker-driven prediction of non-responders facilitates precision medicine for HCC patients. To identify noninvasive circulating biomarkers predicting therapeutic response of Atezo/Bev, we performed simultaneous measurement of 34 plasma proteins and found that plasma IL-6 level was a significant predictor of non-responder for Atezo/Bev therapy. We subsequently confirmed that the progression-free survival and overall survival of the IL-6-high group were significantly shorter than those of the IL-6-low group. In conclusion, circulating IL-6 levels are a novel prognostic biomarker for advanced HCC patients who undergo combined immunotherapy.

**Abstract:**

Atezolizumab/bevacizumab (Atezo/Bev) combination therapy has become a front-line therapy for advanced hepatocellular carcinoma (HCC), but approximately 20% of patients are nonresponders. We investigated circulating biomarkers to predict therapeutic outcomes. We performed simultaneous measurement of 34 proteins using a multiplex bead-based immunoassay in baseline plasma from 34 patients who underwent Atezo/Bev therapy as first- or second-line treatment. Logistic regression analysis showed that plasma IL-6 and interferon alpha (IFNα) levels were significant predictors of non-responders (odds ratio of 13.33 and FDR *p* = 0.021 for IL-6 and IFNα). The progression-free survival (PFS) and overall survival (OS) of patients with high IL-6 levels were significantly shorter than those of patients with low IL-6 levels. Next, we measured baseline plasma IL-6 levels in 64 HCC patients who underwent Atezo/Bev therapy by ELISA. The IL-6-high group showed higher female ratio, AST levels, tumor markers, Child–Pugh score, and vascular invasion ratio. The PFS and OS of the IL-6-high group were significantly shorter than those of the IL-6-low group. Multivariate Cox proportional hazards analysis showed that IL-6 level and age were independent risk factors for disease progression (hazard ratio of 2.785 and *p* = 0.015 for IL-6, and hazard ratio 0.306 and *p* = 0.03 for age). In conclusion, circulating IL-6 levels are a novel prognostic biomarker for advanced HCC patients who undergo combined immunotherapy.

## 1. Introduction

Hepatocellular carcinoma (HCC) is a major cause of cancer death worldwide [1]. Due to its high recurrence rate, many HCC patients progress to an advanced stage and need systemic therapy. The chemotherapy option for advanced HCC was only sorafenib for a decade. However, multiple tyrosine kinase inhibitors, including lenvatinib [2], regorafenib [3], and cabozantinib [4], have been recently developed and have become treatment choices. In 2020, the combination therapy of anti-PD-L1 and anti-VEGF antibodies using atezolizumab and bevacizumab (Atezo/Bev) was approved on the basis of the IMbrave150 trial results, showing significantly better overall survival (OS), progression-free survival (PFS), and quality of life (QoL) than sorafenib treatment [5]. Furthermore, in a recently published update analysis with a median follow-up of 15.6 months, Atezo/Bev therapy showed a 5.8 month longer median OS than sorafenib [6] and a safety profile consistent with the primary analysis [5]. Objective response occurred in 30% of patients treated with Atezo/Bev therapy, and 74% of patients obtained disease control, so this therapy now serves as a potent front-line chemotherapy [7]. On the other hand, 19% of treated patients were reported to be non-responders [5]. When the six total chemotherapy regimens available for advanced HCC are considered [8], it is found that biomarker-driven prediction of non-responders may help precision therapy for each patient.

Although tumor tissue-based efficacy biomarkers of immune checkpoint inhibitors (ICIs), such as tumor-infiltrating lymphocyte counts and PD-1/PD-L1 expression, have been reported in a variety of cancer types [9,10], there is no reliable biomarker for predicting the efficacy of combination immunotherapy in HCC. Furthermore, because advanced HCC patients who are eligible for immunotherapy are often diagnosed with imaging tests without tumor biopsy, it is important to develop noninvasive blood-based biomarkers. Here, utilizing multiplex bead-based immunoassay technology, we simultaneously profiled 34 plasma proteins in the baseline blood of advanced HCC patients who underwent Atezo/Bev therapy and sought to identify biomarkers to predict the patient outcome of this therapy.

## 2. Materials and Methods

### 2.1. Patients and Study Design

Advanced HCC patients from 12 institutions participating in the Osaka Liver Forum (OLF) study group were prospectively registered and underwent Atezo/Bev treatment. Their baseline blood and clinical information were collected. A total of 64 patients were enrolled between November 2020 and May 2021. HCC was diagnosed with dynamic contrast-enhanced CT or contrast-enhanced MRI. Patients underwent Atezo/Bev treatment every three weeks, and the therapy response was evaluated by the guidelines of the modified Response Evaluation Criteria in Solid Tumors (mRECIST) using contrast-enhanced CT or contrast-enhanced MRI [11]. The clinical and biochemical characteristics of the patients were collected at initiation of the treatment. Observation also started from initiation of the treatment. Overall response rate (ORR) was defined as the proportion of patients who achieved complete response (CR) or partial response (PR) as their best overall response according to mRECIST criteria. Disease control rate (DCR) was defined as the proportion of patients who achieved CR, PR, or stable disease (SD) as their best overall response according to mRECIST criteria. Patients were divided into the progressive disease group (PD) or the non-progressive disease group (non-PD) including CR/PR/SD by the initial response to the atezolizumab and bevacizumab therapy. ALBI score was calculated as the following formula: ALBI score = (log10 bilirubin [µmol/L] × 0.66) + (albumin [g/L] × −0.0852) [12]. Plasma from preliminary selected 34 patients who underwent Atezo/Bev therapy as first- or second-line treatment and 5 healthy volunteers was used for multiplex bead-based immunoassay, and plasma from all 64 patients was used for ELISA.

### 2.2. Quantitative Measurement of Multiple Plasma Proteins Using a Multiplex Bead-Based Immunoassay

A total of 34 plasma proteins, including CCL2, CCL3, CCL4, CCL7, CCL19, CX3CL1, CXCL1, CXCL2, CXCL10, DKK1, Fas Ligand, Fas Receptor, GDF15, Granzyme B, HGF, IFNα, IFNγ, IL1β, IL2, IL4, IL5, IL-6, IL7, IL8, IL10, IL12, IL18, MICA, PD-L1, TIE2, TNFα, TSP2, VEGF, and VEGF-C, were measured with a Luminex assay human premixed multianalyte kit (R&D Systems, Minneapolis, MN, USA) according to the manufacturer’s protocol. The MFI was obtained with the Luminex system, and the data were analyzed with Analyst.

### 2.3. Quantitative Measurement of Plasma IL-6 Levels by Enzyme-Linked Immunosorbent Assay (ELISA)

The plasma of HCC patients was stored in a −80 °C deep freezer and analyzed with a Human IL-6 ELISA kit (D6050, R&D Systems) according to the manufacturer’s protocol.

### 2.4. Statistical Analysis

Mann–Whitney *U* tests were used to assess differences between unpaired groups with a nonparametric distribution. One-way analysis of variance (ANOVA) followed by the Kruskal–Wallis test was used for nonparametric multiple comparisons. Chi-squared tests or Fisher’s exact tests were used to analyze categorical data. The Pearson product-moment correlation coefficient was used for the assessment of correlations. In the survival analysis, the end point of OS was defined as the time from the day of treatment initiation until death from any cause. PFS was determined as the time from the day of treatment initiation until disease progression assessed by mRECIST1.1 or death, whichever occurred first. Differences in OS and PFS were analyzed by the Kaplan–Meier method and log-rank test. Factors associated with improved PFS were analyzed using univariate and multivariate Cox proportional hazards regression models. When dichotomizing factors, we used each median value as the cut-off value. Otherwise, the statistical analyses used are indicated in the figure legends. A *p*-value < 0.05 was considered to indicate statistical significance. Prism ver.8.4.2 for Windows (GraphPad Prism, RRID:SCR_002798, San Diego, CA, USA) and JMP^®^ 13 (SAS Institute Inc. RRID:SCR_014242, Cary, NC, USA) were used for the analyses.

## 3. Results

### 3.1. Quantitative Multiplex Measurement of Plasma Proteins Showed That High Baseline IL-6 Levels Were Associated with Poor Treatment Response in HCC Patients Who Underwent Atezo/Bev Therapy

#### 3.1.1. Quantitative Multiplex Measurement of Plasma Proteins

**Figure 1:**
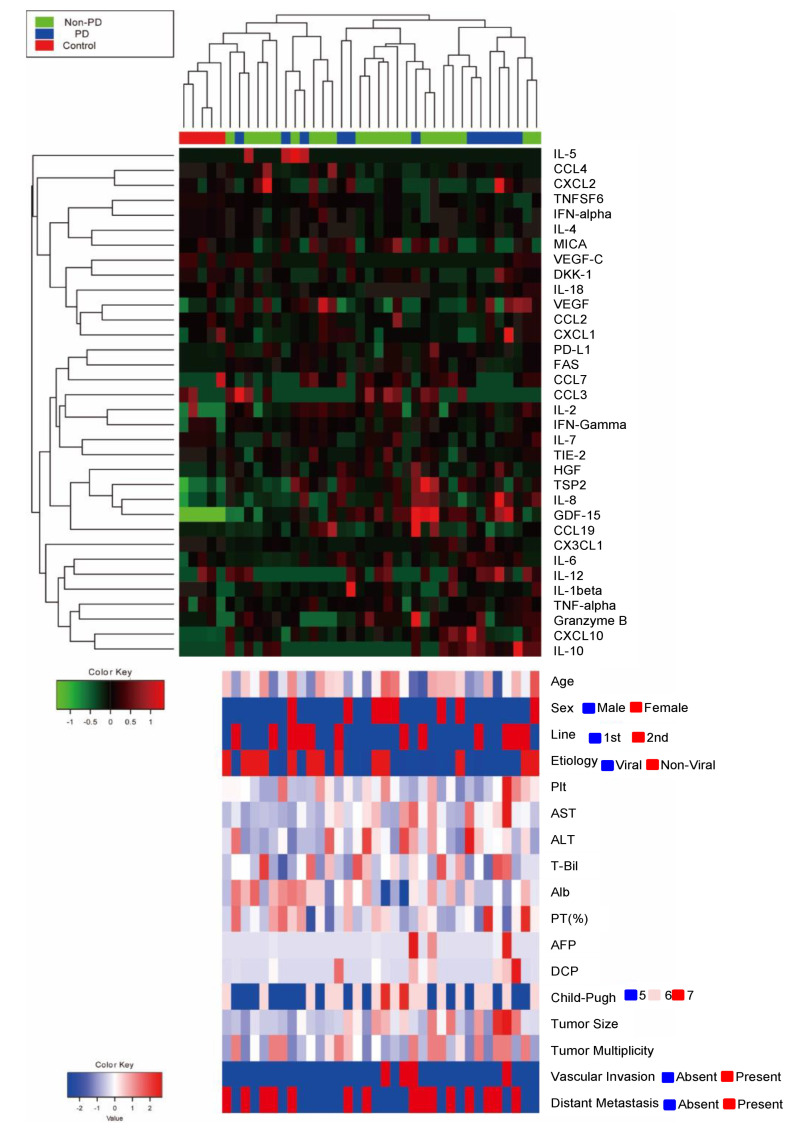
Heatmap of 34 plasma proteins quantified by a multiplex bead-based immunoassay using the baseline plasma of 34 HCC patients treated with Atezo/Bev therapy and 5 healthy controls. The upper heatmap shows each protein level, and the bottom heatmap shows patient backgrounds. IL-5, interleukin-5; CCL4, CC motif chemokine 4; CXCL2, C-X-C motif ligand 2; TNFSF6, tumor necrosis factor superfamily 6; IFN-alpha, interferon-alpha; IL-4, interleukin-4; MICA, MHC class I polypeptide-related sequence A; VEGF-C, vascular endothelial growth factor-C; DKK-1, dickkopf related protein-1; IL-18, interleukin-18; VEGF, vascular endothelial growth factor; CCL2, CC motif chemokine 2; CXCL1: C-X-C motif ligand 1; PD-L1, programmed death-ligand 1; CCL7: CC motif chemokine 7; CCL3, CC motif chemokine 3; IL-2: interleukin-2; IFN-Gamma: interferon-gamma; IL-7, interleukin-7; HGF, hepatocyte growth factor; TSP2, thrombospondin 2; IL-8, interleukin-8; GDF-15, growth/differentiation factor-15; CCL19, CC motif chemokine 19; CX3CL1, C-X3-C motif chemokine ligand 1; IL-6, interleukin-6; IL-12: interleukin-12; IL-1beta, interleukin-1 beta; TNF-alpha, tumor necrosis factor-alpha; CXCL10, C-X-C motif ligand 10; IL-10, interleukin-10; Plt, platelet cell count; AST, aspartate aminotransferase; ALT, alanine aminotransferase; T-Bil, total bilirubin; Alb, albumin; PT, prothrombin time; AFP, alpha fetoprotein; DCP, Des-gamma-carboxy-prothrombin; PD, progressive disease; non-PD, non-progressive disease including complete response, partial response, and stable disease.

To search for blood-based biomarkers for predicting the efficacy of Atezo/Bev therapy for advanced HCC patients, we first performed simultaneous measurement of 34 plasma proteins using a multiplex bead-based immunoassay in baseline plasma from preliminary selected 34 patients who underwent Atezo/Bev therapy as first- or second-line treatment and five healthy controls. The clinical characteristics of HCC patients are shown in Table S1. The ORR and DCR evaluated by mRECIST version 1.1 were 26.5% and 64.7%, respectively (CR/PR/SD/PD: 3/6/13/12) (Table S1, Figure S1A). The median PFS was 187 days (Figure S1B). Four patients died of HCC during the observation time (Figure S1C). Hierarchical clustering based on the plasma cytokine levels clearly distinguished HCC patients from healthy controls but failed to classify HCC patients on the basis of the initial response to Atezo/Bev therapy (Figure 1).

#### 3.1.2. Logistic Regression Analysis of Factors Related to Progressive Disease

We first analyzed the association of tumor malignancy and each plasma protein level. The levels of GDF15, CCL19, and granzyme B showed a strong positive association with serum AFP levels (r^2^ > 0.6) (Table S2). Therefore, these proteins might be potential candidate biomarkers of tumor malignancy in HCC patients. We then searched for plasma proteins associated with the progressive disease (PD) at the initial evaluation upon Atezo/Bev therapy. Among 34 proteins, logistic regression analysis showed that plasma IL-6 and IFNα levels were significant predictors of initial PD (Table 1).

#### 3.1.3. PFS and OS According to Plasma IL-6 Levels

**Figure 2:**
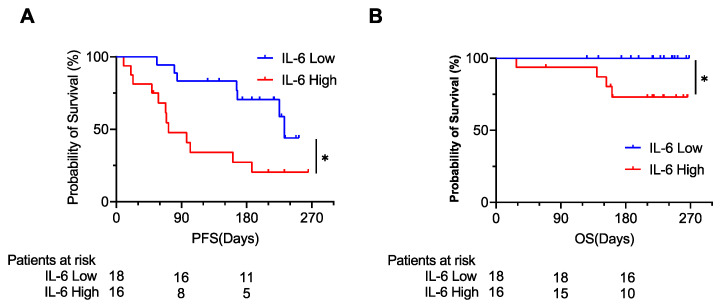
The Kaplan–Meier curve for the progression-free survival (PFS) (**A**) and overall survival (OS) (**B**). The patients were divided into 2 groups by the median value of plasma IL-6. * *p* < 0.05.

The PFS and OS of patients with high IL-6 levels were significantly shorter than those of patients with low IL-6 levels (Figure 2A,B). On the other hand, although the PFS and OS of patients with high IFNα levels were shorter than those of patients with low IFNα levels (Figure S2A,B), the difference of the PFS was not statistically significant.

### 3.2. High Baseline Plasma IL-6 Levels Were an Independent Predictor of Poor PFS in HCC Patients during Atezo/Bev Therapy

#### 3.2.1. Patient Characteristics of the Validation Cohort

To individually validate our multiplex assay, we quantitatively measured the baseline plasma IL-6 levels of all 64 HCC patients who underwent Atezo/Bev therapy by ELISA. The patient clinical background is shown in Table 2 and Table S3. The median age was 75 years, and the percentage of males was 78.1%. Thirty-six patients underwent Atezo/Bev therapy as the first-line setting, while 28 patients underwent Atezo/Bev therapy as the later-line setting. All but four patients had Child–Pugh A, and the median ALBI score was −2.435. The median AFP and DCP levels were 11 ng/mL and 276 mAU/mL, respectively. The numbers of patients diagnosed with Barcelona Clinic Liver Cancer (BCLC) stages A, B, and C were 1, 29, and 34, respectively. The median observation time after the initiation of Atezo/Bev therapy was 104 days.

#### 3.2.2. Treatment Response and Kaplan–Meier Curves of PFS and OS

**Figure 3:**
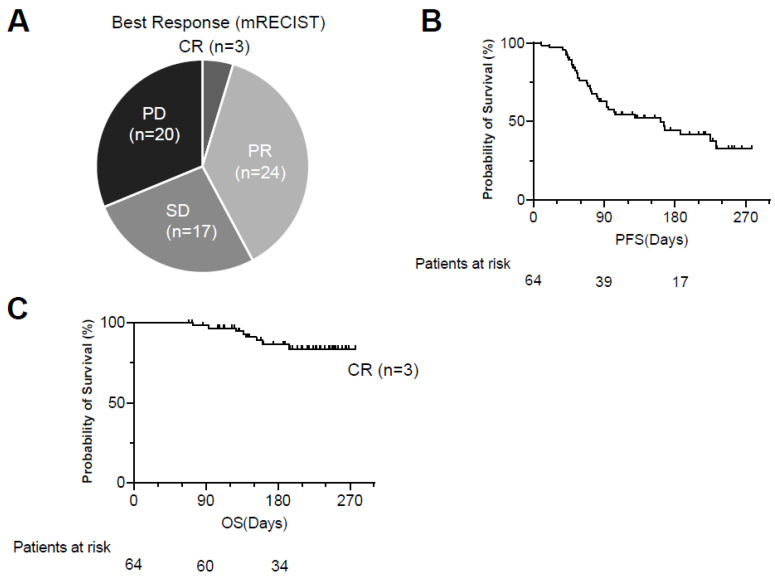
Treatment response to Atezo/Bev therapy. (**A**) The best response to Atezo/Bev was evaluated by mRECIST. (**B**,**C**) Kaplan–Meier curves of progression-free survival (PFS) (**B**) and overall survival (OS) (**C**). Atezo/Bev, Atezolizumab and bevacizumab; CR, complete response; PR, partial response; SD, stable disease; PD, progressing disease.

The ORR and DCR evaluated by mRECIST were 42.2% and 68.8%, respectively (CR/PR/SD/PD: 3/24/17/20) (Figure 3A). The cumulative PFS rates at 90 days, 180 days, and 270 days were 62.7%, 44.4%, and 32.8%, respectively, and the median PFS was 161 days (Figure 3B). Eight patients died of HCC during the observation time (Figure 3C).

#### 3.2.3. Patient Characteristics According to the Plasma IL-6 Levels

We first confirmed the linear correlation between the plasma IL-6 levels examined by ELISA and the bead-based Luminex assay (Figure S3). Then, the patients were divided into two groups on the basis of the median plasma IL-6 level measured by ELISA (IL-6 high vs. IL-6 low). The IL-6-high group showed a higher female ratio; AST, AFP, and DCP levels; Child–Pugh score; and vascular invasion ratio (Table 3).

#### 3.2.4. Treatment Response and Kaplan–Meier Curves of PFS and OS According to Plasma IL-6 Levels

**Figure 4:**
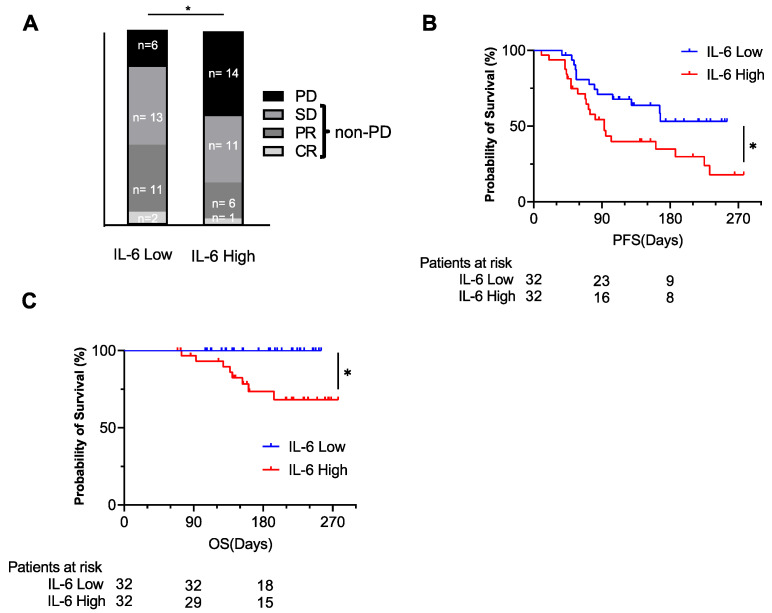
The baseline plasma IL-6 level was examined by ELISA. The patients were divided into 2 groups by the median value of plasma IL-6. The best response to the treatment in each group (**A**) (* *p* < 0.05 by chi-squared test, PD vs. non-PD) and the Kaplan–Meier curves of PFS (**B**) and OS (**C**) for each group (* *p* < 0.05). CR, complete response; PR, partial response; SD, stable disease; PD, progressing disease; non-PD, non-progressive disease including complete response, partial response, and stable disease.

The ORR and DCR were 40.6% and 81.3% in the IL-6-low group (CR/PR/SD/PD: 2/11/13/6) and 21.9% and 56.3% in the IL-6-high group (CR/PR/SD/PD: 1/6/11/14), respectively (Figure 4A). The PD ratio at the initial evaluation was significantly higher in the IL-6-high group compared to the IL-6-low group (Figure 4A). The PFS and OS of the IL-6-high group were significantly shorter than those of the IL-6-low group (Figure 4B,C).

#### 3.2.5. Univariate and Multivariate Cox Proportional Hazards Analysis of Factors Related to PFS

Univariate Cox proportional hazards analysis showed that later-line treatment, younger age, and high AST and IL-6 levels were significantly associated with poor PFS (Table 4). Among these four variables, multivariate Cox proportional hazards analysis showed that age and IL-6 level were independent risk factors for disease progression in HCC patients who underwent Atezo/Bev therapy (Table 4). The similar results were also observed when factors were analyzed as continuous value (Table S4). Taken together, these findings suggest that circulating IL-6 levels may be a novel prognostic biomarker for advanced HCC patients who undergo combined immunotherapy.

## 4. Discussion

In the present study, we profiled a variety of plasma proteins in advanced HCC patients who underwent Atezo/Bev therapy and found a novel association between baseline plasma IL-6 levels and poor prognosis. IL-6 is a proinflammatory cytokine, and its expression is induced in a variety of acute or chronic inflammatory conditions [13]. IL-6 is known to be involved in various liver pathologies, especially liver regeneration and cancer [14]. IL-6 induced compensatory proliferation of hepatocytes in a diethylnitrosamine (DEN)-induced hepatocarcinogenesis model, and either deletion of IL-6 or gp130, a second receptor protein associated with the IL-6 receptor, suppressed liver tumor development in a DEN model in mice [15]. IL-6 also promoted tumor progression via STAT3 signaling in an obesity-induced liver tumor mouse model [16]. This experimental evidence suggests an oncogenic role of IL-6 in HCC. Clinically, a recent meta-analysis of 18 studies including approximately 1000 HCC and hepatitis patients showed stepwise elevation of serum IL-6 levels according to the disease stage from healthy to hepatitis and cirrhosis and to HCC [17]. Moreover, serum IL-6 levels are positively correlated with the clinical stage of HCC patients and could predict the early recurrence of HBV-HCC after curative resection, suggesting the high malignant potential of HCC patients with high serum IL-6 levels [18,19]. In the present study, we also showed that HCC patients with high plasma IL-6 levels had higher AFP and DCP levels and a higher ratio of macrovascular invasion than those with low IL-6 levels (Table 3), suggesting a positive association between circulating IL-6 levels and HCC disease progression.

Multivariate analysis using the Cox proportional hazards model demonstrated that high levels of baseline plasma IL-6 were predictors of shorter PFS in HCC patients who underwent Atezo/Bev therapy, independent of disease stage and liver function. IL-6 is reported to play both promoting and suppressing roles in tumor immunity. For tumoricidal roles, IL-6 mediates chemokines and induces T cell infiltration [20]. On the other hand, IL-6 recruits myeloid-derived suppressor cells (MDSCs), which inhibit T cells reactive to tumor antigen, to the tumor site in many types of cancer, including HCC [21,22,23]. MDSCs are indeed known to hinder the anticancer activity of ICIs [24]. It has also been shown that the high baseline serum IL-6 levels of melanoma patients were associated with poor response after nivolumab or ipilimumab treatment [25]. Thus, it may be interesting to further study the possible interaction between IL-6 signaling and ICI efficacy in HCC patients.

Among a variety of plasma proteins measured by the multiplex assay, some were found to be strongly correlated with the disease status of HCC. GDF15 was most strongly associated with AFP levels (Table S2). In a previous study, we showed that the serum GDF15 level is positively correlated with the clinical stage of HCC and reflects prognosis [26]. We also demonstrated the oncogenic role of GDF15 from hepatic stellate cells in HCC [26]. Therefore, the results of our current study further strengthen the importance and utility of GDF15 as a biomarker of HCC malignancy.

Regarding other liquid biopsy biomarkers for the efficacy of immunotherapy, genetic alterations affect the immune cell infiltration pattern and thus might affect the efficacy of immunotherapy [27]. The WNT/CTNNB1 signaling pathway was recently reported to suppress immune cell infiltration [28,29], and this signaling activation in the tumor site was negatively associated with ICI treatment in HCC [30]. In the present study, we analyzed the plasma levels of DKK1, which is an antagonist of WNT signaling, as a potential surrogate marker of WNT/CTNNB1 signaling activity, but they were not associated with the response to Atezo/Bev therapy.

There are several limitations for this study. First, the cohort size of our study is relatively small. One possible reason is that the time since the Atezo/Bev therapy was approved is still as short as about a year. Second, although this is a multicenter study, all the participants were Japanese, and thus there was no rational diversity. Lastly, the observation period was not long enough to appropriately evaluate overall survival.

## 5. Conclusions

Through the multiplex measurement of plasma proteins, we identified and validated the fact that circulating IL-6 levels are a novel biomarker for predicting the prognosis of advanced HCC patients who underwent combined immunotherapy.

## Figures and Tables

**Table 1:** Logistic regression analysis of factors related to progressive disease.

Variable	Cut Off	ODDs Ratio	95% CI	FDR *p* Value
CCL2	>288/288	0.595	0.144–2.467	0.642
CCL3	Detected/Not detected	0.200	0.035–1.132	0.405
CCL4	>220/220	0.595	0.144–2.467	0.642
CCL7	Detected/Not detected	0.900	0.139–5.811	1.000
CCL19	>80/80	0.595	0.144–2.467	0.642
CX3CL1	>900/900	1.680	0.4054–6.962	0.642
CXCL1	>120/120	2.889	0.664–12.57	0.504
CXCL2	>145/145	0.595	0.144–2.467	0.642
CXCL10	>56/56	0.346	0.080–1.507	0.504
DKK1	>1000/1000	1.680	0.405–6.962	0.642
Fas Ligand	>45/45	1.000	0.245–4.08	1.000
Fas Receptor	>8400/8400	0.595	0.144–2.47	0.642
GDF15	>2400/2400	1.000	0.245–4.083	1.000
Granzyme B	>9/9	1.000	0.245–4.083	1.000
HGF	>100/100	1.000	0.245–4.083	1.000
IFNα	>2.1/2.1	13.330	2.24–79.44	0.021
IFNγ	>11.05/11.05	5.250	1.093–25.21	0.323
IL1β	>3.06/3.06	2.889	0.663–12.57	0.504
IL2	>6.9/6.9	1.400	0.339–5.79	0.807
IL4	>49/49	3.500	0.795–15.40	0.502
IL5	Detected/Not detected	2.000	0.244–16.36	0.681
IL6	>3.2/3.2	13.333	2.234–79.438	0.021
IL7	>2.4/2.4	0.595	0.144–2.467	0.642
IL8	>17/17	1.680	0.405–6.962	0.642
IL10	Detected/Not detected	2.022	0.475–8.434	0.642
IL12	Detected/Not detected	1.680	0.405–6.962	0.642
IL18	>260/260	1.680	0.405–6.962	0.642
MICA	>55/55	1.000	0.245–4.08	1.000
PD-L1	>18/18	0.346	0.080–1.507	0.504
TIE2	>15,300/15,300	0.286	0.065–1.257	0.502
TNFα	>5/5	0.595	0.137–2.445	0.642
TSP2	>43,000/43,000	1.679	0.405–6.962	0.642
VEGF	>30/30	1.680	0.405–6.962	0.642
VEGF-C	>480/480	1.000	0.245–4.08	1.000

**Table 2:** Patient characteristics (*N* = 64).

Characteristic	Unit	Value (Median, IQR)
Age	Years	75 (63–79)
Sex	Male/Female	50/14
Etiology	Non-viral/Viral	25/39
Platelets	×10^4^/μL	13.8 (16.1–11.1)
Total Bilirubin	mg/dL	0.7 (0.5–1)
AST	U/L	37 (24–51)
ALT	U/L	26 (17–35)
PT	%	93 (82–101)
Albumin	g/dL	3.7 (3.3–4.0)
Child-Pugh Score	5/6/7	34/26/4
AFP	ng/mL	11 (3.1–200)
DCP	mAU/mL	276 (53–1544)
Distant Metastasis	Present/Absent	31/33
Vascular Invasion	Present/Absent	7/57
BCLC Stage	A,B/C	30/34
ALBI Score		−2.435
Tratment Line	1st/2nd/3rd/4th-	36/17/6/5
Observation Time	Days	104 (56–184)

Abbreviations: AST, aspartate transaminase; ALT, alanine aminotransferase; PT, prothrombin time; AFP, alpha-fetoprotein; DCP, des-γ-carboxy prothrombin; BCLC, Barcelona clinic liver cancer.

**Table 3:** Patients’ characteristics according to the plasma IL-6 levels.

Characteristic	Unit	Value (Median, IQR)		*p* value
		IL6 High (N=32)	IL6 Low (*N* = 32)	
Age	Years Old	75 (67–79)	72 (61–81)	0.819
Sex	Male/Female	21/11	29/3	0.032
Etiology	Non-viral/Viral	12/20	14/18	0.984
Platelets	×10^4^/μL	13.3 (9.0–17.8)	14.5 (12.6–16.1)	0.163
Total Bilirubin	mg/dL	0.7 (0.6–1.1)	0.8 (0.5–0.9)	0.995
AST	U/L	45 (29–57 )	29 (24–42)	0.014
ALT	U/L	30 (19–47)	22 (16–33)	0.237
PT	%	93 (82–100)	91 (82–103)	0.767
Albumin	g/dL	3.6 (3.3–3.9)	3.9 (3.5–4.1)	0.077
Child-Pugh Score	5/6/7	14/14/4	20/12/0	0.041
AFP	ng/mL	57 (6.3–3718)	6.6 (3–86)	0.011
DCP	mAU/mL	440 (102–9982)	108 (39–598)	0.018
Distant Metastasis	Present/Absent	16/16	15/17	0.803
Vascular Invasion	Present/Absent	7/25	0/32	0.001
BCLC Stage	A,B/C	13/19	17/15	0.316
Tratment Line	1st/2nd/3rd/4th-	16/9/4/3	20/8/2/2	0.709
Observation Time	Days	84 (49–160)	130 (75–195)	0.079

Abbreviations: AST, aspartate transaminase; ALT, alanine aminotransferase; PT, prothrombin time; AFP, alpha-fetoprotein; DCP, des-γ-carboxy prothrombin; BCLC, Barcelona clinic liver cancer.

**Table 4:** Cox proportional hazards analysis of factors related to PFS.

		Univariate Analysis			Multivariate Analysis		
Characteristic	Unit	Hazard Ratio	95% CI	*p* Value	Hazard Ratio	95% CI	*p* Value
Age	>72/72	0.432	0.220–0.849	0.015	0.306	0.140–0.668	0.003
Sex	Male/Femal	1.284	0.571–2.887	0.545			
Etiology	Viral/Non-viral	1.028	0.516–2.048	0.938			
Platelets	>12/12	1.134	0.578–2.230	0.712			
Total Bilirubin	>0.8/0.8	1.011	0.519–1.968	0.975			
AST	>40/40	2.58	1.289–5.163	0.008	1.655	0.785–3.488	0.186
ALT	>27/27	1.77	0.894–3.524	0.101			
PT	>90/90	1.544	0.754–3.167	0.235			
Albumin	>3.6/3.6	1.148	0.583–2.260	0.689			
Child-Pugh Score	6,7/5	0.76	0.386–1.497	0.428			
AFP	>11/11	1.953	0.998–3.820	0.051			
DCP	>276/276	1.478	0.742–2.941	0.266			
Distant Metastasis	Present/Absent	1.349	0.693–2.627	0.378			
Vascular Invasion	Present/Absent	1.924	0.792–4.67	0.148			
BCLC Stage	C/A,B	1.241	0.636–2.426	0.526			
Treatment Line	1st/later	0.445	0.227–0.873	0.019	0.661	0.334–1.310	0.236
IL6	>4.77/4.77	2.197	1.104–4.372	0.025	2.785	1.216–6.380	0.015

Abbreviations: AST, aspartate transaminase; ALT, alanine aminotransferase, PT, prothrombin time; AFP, alpha-fetoprotein; DCP, des-γ-carboxy prothrombin; BCLC, Barcelona clinic liver cancer.

## Data Availability

The data presented in this study are available on request from the corresponding author.

## Supplementary Materials

The following are available online at https://www.mdpi.com/article/10.3390/cancers14040883/s1, Figure S1: The initial response to Atezo/Bev and Kaplan–Meier curves of PFS and OS. Figure S2: The Kaplan–Meier curves for the PFS and OS stratified by IFNα levels. Figure S3: The plasma IL-6 levels examined by ELISA and the bead-based Luminex assay. Table S1: Patient characteristics. Table S2: Correlation between each plasma protein and AFP level. Table S3: Comparison of patient characteristics. Table S4: Cox hazard analysis for progressing disease.
