# Supplementary material for: Interleukin-6 Is a Circulating Prognostic Biomarker for Hepatocellular Carcinoma Patients Treated with Combined Immunotherapy
Source: Cancers (Basel). 2022 Feb 10;14(4):883. doi: 10.3390/cancers14040883 (PMC8870238; doi:10.3390/cancers14040883)
Supplement: Supplementary file 1 [file cancers-14-00883-s001.zip › cancers-1570345-supplementary.pdf]

Supplementary Figure S1

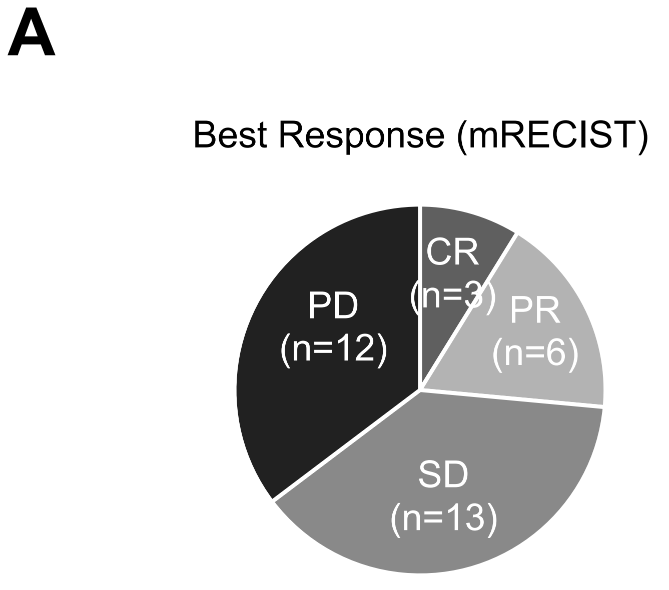

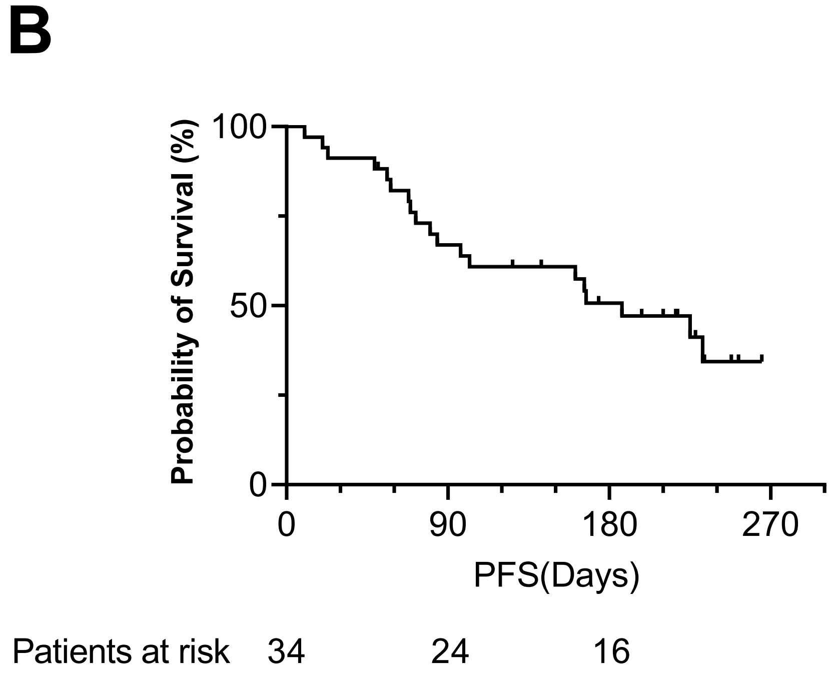

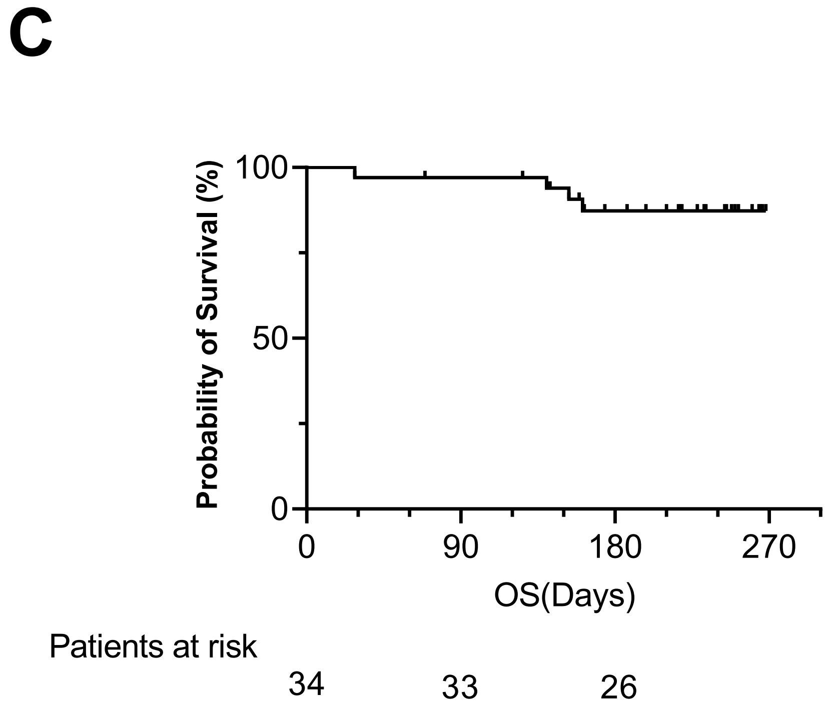

Figure S1. A total of 34 HCC patients were treated with Atezo/Bev therapy. **(A)** The initial response to Atezo/Bev was evaluated by mRECIST. (**B, C**) Kaplan–Meier curves of progression-free survival (PFS) **(B)** and overall survival (OS) **(C)**. Atezo/Bev; Atezolizumab and bevacizumab, CR; complete response, PR; partial response, SD; stable disease, PD; progressing disease.

Supplementary Figure S2

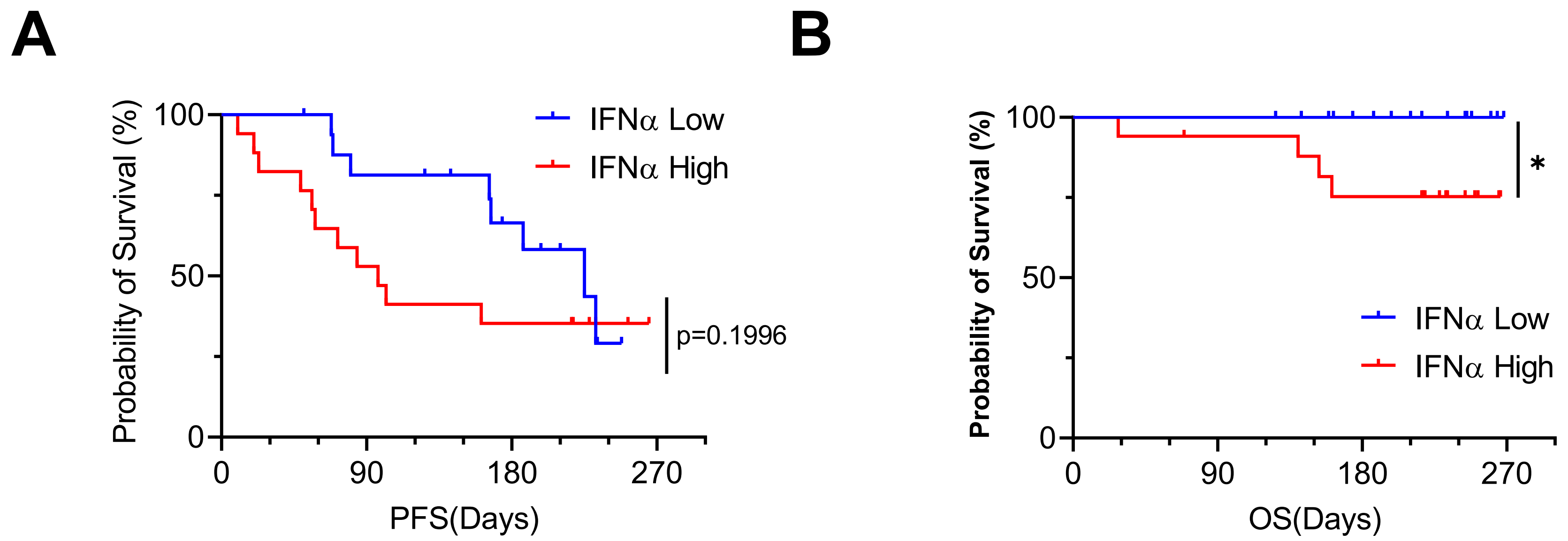

Patients at risk

	0	90	180
IFNα Low	17	14	9
IFNα High	17	10	7

Patients at risk

	0	90	180
IFNα Low	17	17	13
IFNα High	17	16	13

Figure S2. **(A, B)** The Kaplan–Meier curves for the progression-free survival (PFS) **(A)** and overall survival (OS) **(B)** . The patients were divided into 2 groups by the median value of plasma IFNα. *:p<0.05.

Supplementary Figure S3

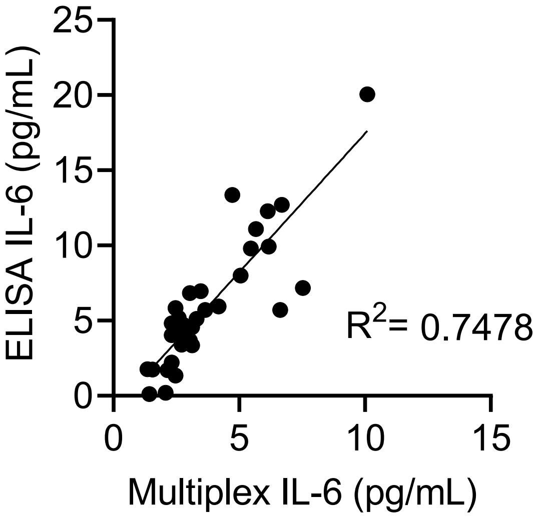

Figure S3. The pretreatment plasma IL-6 level of 34 HCC patients was examined with ELISA and Luminex assay. Their correlation was analyzed with simple linear regression.

Table S1. Patient characteristics(N=34).

Characteristic	Unit	Value (Median, IQR)
Age	Years	73 (61-79)
Sex	Male/Female	26/8
Etiology	HBV/HCV/NBNC	11/10/13
Platelets	$\times 10^4/\mu L$	12.3 (8.9-14.9)
Total Bilirubin	mg/dL	0.85 (0.6-1.1)
AST	U/L	41 (27-53)
ALT	U/L	29 (19-37)
PT	%	92 (84-101)
Albumin	g/dL	3.6 (3.3-3.9)
Child-Pugh Score	5/6/7	16/16/2
AFP	ng/mL	32 (5.5-251)
DCP	mAU/mL	237 (48-4529)
Distant Metastasis	Present/Absent	15/19
Vascular Invasion	Present/Absent	4/30
BCLC Stage	A/B/C	1/15/18
Observation Time	Days	106 (61-147)
Tratment Line	1st/2nd	22/12
Initial Response (mRECIST)	CR/PR/SD/PD	1/6/15/12
Best Response (mRECIST)	CR/PR/SD/PD	3/6/13/12

Abbreviations: AST, aspartate transaminase; ALT, alanine aminotransferase; PT, prothrombin time AFP, alpha-fetoprotein; BCLC, Barcelona clinic liver cancer; DCP, des-γ-carboxy prothrombin mRECIST:Modified Response Evaluation Criteria in Solid Tumors

Table S2. Correlation between each plasma protein and AFP level.

Proteins	R squared	FDR adjusted P value
GDF15	0.82	<0.0001
CCL19	0.73	<0.0001
GranzymeB	0.68	<0.0001
HGF	0.46	0.001
IL18	0.26	0.026
CX3CL1	0.25	0.030
TSP2	0.22	0.036
CXCL1	0.22	0.036
IFNγ	0.18	0.090
DKK1	0.17	0.094
IL8	0.14	0.153
TNFα	0.14	0.165
TIE2	0.13	0.181
MICA	0.12	0.182
IL6	0.08	0.341
CCL7	0.07	0.405
IFNα	0.05	0.496
Fas ligand	0.05	0.530
IL4	0.04	0.546
CXCL10	0.02	0.722
PD-L1	0.02	0.762
Fas receptor	0.02	0.762
CCL3	0.01	0.805
VEGF	0.01	0.805
IL5	0.01	0.805
IL12	0.01	0.821
VEGF-C	0.00	0.871
IL10	0.00	0.912
CCL4	0.00	0.911
IL1b	0.00	0.911
IL7	0.00	0.911
IL2	0.00	0.922
CCL2	0.00	0.923
CXCL2	0.00	0.991

Table S3. Comparison of patient characteristics (Median, IQR).

Characteristic	Unit	Cohort for multiplex bead-based immunoassay(N=34)	Cohort for ELISA(N=64)	p value
Age	Years	73 (61-79)	75 (63-79)	0.811
Sex	Male/Female	26/8	50/14	0.852
Etiology	HBV/HCV/NBNC	11/10/13	15/24/25	0.705
Platelets	$\times 10^4/\mu L$	12.3 (8.9-14.9)	13.8 (11.1-16.1)	0.361
Total Bilirubin	mg/dL	0.85 (0.6-1.1)	0.7 (0.5-1.0)	0.239
AST	U/L	41 (27-53)	37 (24-52)	0.455
ALT	U/L	29 (19-37)	26 (17-35)	0.604
PT	%	92 (84-101)	93 (82-102)	0.898
Albumin	g/dL	3.6 (3.3-3.9)	3.7 (3.3-4.0)	0.629
Child-Pugh Score	5/6/7	16/16/2	34/26/4	0.828
AFP	ng/mL	32 (5.5-251)	11 (3.2-200)	0.573
DCP	mAU/mL	237 (48-4529)	276 (53-1544)	0.945
Distant Metastasis	Present/Absent	15/19	31/33	0.683
Vascular Invasion	Present/Absent	4/30	7/57	0.902
BCLC Stage	A/B/C	1/15/18	5/25/34	0.101
Tratment Line	1st/2nd/3rd-	22/12	36/17/11	0.015
Initial Response mRECIST	CR/PR/SD/PD	1/6/15/12	1/16/19/26	0.798

Abbreviations: AST, aspartate transaminase; ALT, alanine aminotransferase; PT, prothrombin time
AFP, alpha-fetoprotein; BCLC, Barcelona clinic liver cancer; DCP, des-γ-carboxy prothrombin
mRECIST:Modified Response Evaluation Criteria in Solid Tumors

Table S4. Cox Hazard Analysis for progressing disease.

		Univariate Analysis			Multivariate Analysis		
**Characteristic**	**Unit**	**Hazard ratio**	**95% CI**	**p value**	**Hazard ratio**	**95% CI**	**p value**
Age	every1 y.o.	0.964	0.935-0.993	0.016	0.954	0.920-0.989	0.012
Sex	Male/Femal	1.284	0.571-2.887	0.545			
Etiology	Viral/Non-viral	1.028	0.516-2.048	0.938			
Platelets	every 1×104/μL	0.999	0.949-1.042	0.990			
Total Bilirubin	every 1mg/dL	0.796	0.280-2.032	0.650			
AST	every 1U/L	1.006	0.993-1.017	0.316			
ALT	every 1U/L	1.002	0.985-1.018	0.752			
PT	every 1%	1.016	1.043-0.985	0.247			
Albumin	eery 1g/dL	1.149	0.607-2.221	0.674			
Child-Pugh Score	6,7 / 5	0.76	0.386-1.497	0.428			
AFP	every 1ng/mL	1	0.999-1.000	0.292			
DCP	every 1mAU/mL	1	1.000-1.00004	0.015	1	0.999-1.000	0.105
Distant Metastasis	Present/Absent	1.349	0.693-2.627	0.378			
Vascular Invasion	Present/Absent	1.924	0.792-4.67	0.148			
BCLC Stage	C / A,B	1.241	0.636-2.426	0.526			
Treatment Line	1st / later	0.445	0.227-0.873	0.019	0.652	0.711-3.30	0.273
IL6	every 1 pg/ml	1.052	1.011-1.088	0.016	1.051	1.007-1.091	0.012

Abbreviations: AST, aspartate transaminase; ALT, alanine aminotransferase

PT, prothrombin time; AFP, alpha-fetoprotein; DCP, des-γ-carboxy prothrombin

BCLC, Barcelona clinic liver cancer
